# Multi-scale U-like network with attention mechanism for automatic pancreas segmentation

**DOI:** 10.1371/journal.pone.0252287

**Published:** 2021-05-27

**Authors:** Yingjing Yan, Defu Zhang

**Affiliations:** School of Informatics, Xiamen University, Xiamen, Fujian, China; University of South Carolina, UNITED STATES

## Abstract

In recent years, the rapid development of deep neural networks has made great progress in automatic organ segmentation from abdominal CT scans. However, automatic segmentation for small organs (e.g., the pancreas) is still a challenging task. As an inconspicuous and small organ in the abdomen, the pancreas has a high degree of anatomical variability and is indistinguishable from the surrounding organs and tissues, which usually leads to a very vague boundary. Therefore, the accuracy of pancreatic segmentation is sometimes below satisfaction. In this paper, we propose a 2.5D U-net with an attention mechanism. The proposed network includes 2D convolutional layers and 3D convolutional layers, which means that it requires less computational resources than 3D segmentation models while it can capture more spatial information along the third dimension than 2D segmentation models. Then We use a cascaded framework to increase the accuracy of segmentation results. We evaluate our network on the NIH pancreas dataset and measure the segmentation accuracy by the Dice similarity coefficient (DSC). Experimental results demonstrate a better performance compared with state-of-the-art methods.

## Introduction

Segmenting organs such as the spleen, liver, and pancreas from abdominal CT scans is a critical prerequisite of computer-aided diagnosis (CAD) [1, 2], quantitative and qualitative analysis. In this paper, we are committed to automatic pancreas segmentation, which is an extensively useful CAD technique for the diagnosis and prognosis of pancreatic cancer. So far, automatic segmentation for other organs (e.g., lung, kidney and liver) has reached relatively a high accuracy. However, the pancreas has irregular shapes, great individual differences and vague boundary, while occupying only a very small part (e.g., less than 1%) of the whole CT volumes, which results in the difficulty of segmentation with high accuracy [3, 4].

In recent years, with the fast development of deep learning, natural image semantic models are widely used to complete medical image segmentation tasks, such as full convolution network(FCN) [5] and U-net [6]. Through skip connection, U-net can extract more features from the target tissue and obtain more accurate information than FCN while fusing features in the decoder part. Integrating deep supervision into the network can avoid the risk of gradient vanish in the training process and can effectively improve the performance of the model [7, 8]. Among these complex organ segmentation tasks, pancreas segmentation is one of the most challenging tasks in medical image segmentation [9].

**Fig 1:**
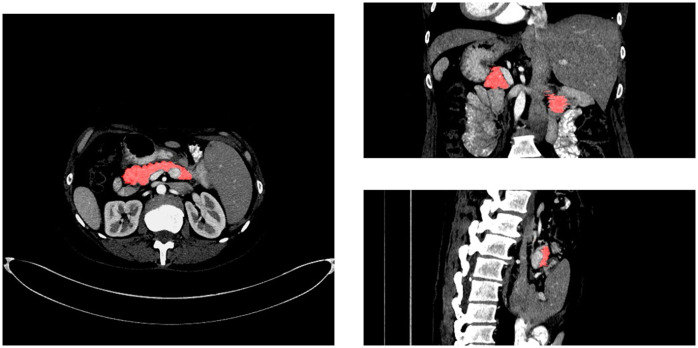
Slices from three different viewpoints. Pancreas is highlighted in red and is a small organ with vague boundary and irregular shape.

In general, we can divide pancreas segmentation methods into two categories, namely methods based on 2D networks and methods based on 3D networks. 3D networks (e.g., 3D U-net [10] and V-net [11]) use the entire CT volume as the input of the network, which can capture 3D spatial information of the CT volume. However, the high requisition of computing resources, especially GPU memory, restricts the depth of the networks and the number of the feature maps, which are two significant factors for performance improvement [12]. In addition, 3D networks are also faced with the problem that there are only a few CT scans available for training. The main reason is that manual labeling requires a lot of time and labor costs. 2D networks use slices by cutting along three axes (the coronal, sagittal and axial views, see Fig 1) from the CT volume. Each slice from three viewpoints is processed separately in the network and prediction results of these viewpoints are fused via majority vote to construct a 3D pancreas volume [13, 14]. However, the pancreas merely occupies less than 2% of the entire CT volume, and its boundary with surrounding tissues and organs is blurred, which means the network may be confused by the background region. To alleviate this problem, a two-stage framework is taken into consideration. In order to reduce the interference of the background region, Zhou et al. [15] proposed a coarse-to-fine framework. Firstly, a 2D FCN is used to segment the slices roughly. Then, according to the results of the coarse segmentation, the pancreatic region is located and the CT volume is clipped to get a smaller block containing the pancreatic region, which is then inputted into another 2D FCN for fine segmentation. Cai et al. [16] presented an end-to-end stacked CNN-RNN segmentation model. The training process is to first train a 2D convolution neural network (CNN) to segment multi-layer adjacent pancreas regions and then the segmentation results are input into a recurrent neural network (RNN). RNN further obtains accurate results by integrating the information of its adjacent layers. Yu et al. [17] used two cascaded 2D FCN to segment the pancreas. First a FCN is used to acquire a coarse segmentation probability map. Then the probability map is transformed to a spatial weight map by a saliency conversion module, and multiplied with the input slice to get a slice containing the coarse segmentation information. According to the coarse segmentation probability map, the entire CT volume is cropped into a smaller volume containing the pancreatic region. Finally, the loss function of the coarse and fine segmentation is optimized.

With the help of attention mechanism, the network can focus on the most relevant features without extra supervision. Therefore, many computer vision tasks have integrated attention mechanism into CNN, including emotion recognition [18], object detection [19], human pose estimation [20] and semantic segmentation [21]. Dual attention network (DANet) [22] applies attention mechanism to both spatial dimension and channel dimension. The output of these two attention modules is fused to get more robust feature expression, so as to obtain more accurate segmentation results. To generate dense and pixel-wise contextual information, these attention modules have to aggregate contextual information of each pixel from all pixels, which will generate a huge feature map, and result in high computational complexity and high occupation of GPU memory. To address these problems, Huang et al. [23] proposed a new cross attention module. The cross attention module merely aggregates contextual information for each position from pixel in horizontal and vertical directions. It can obtain the contextual information from all pixels through two continuous cross attention modules. Compared with the attention modules mentioned above, cross attention module has high computational efficiency and occupies less GPU memory, while achieving outstanding performance. Medical image segmentation tasks also benefit from attention mechanisms. For example, Oktay et al. [24] proposed attention U-net, which can easily integrate attention gates into U-net model with increasing minimal computational resources while improving the segmentation performance.

In this paper, inspired by the excellent performance of U-net and its variants in medical image segmentation, we propose a 2.5D U-like architecture, and apply a dense convolution block with skip connection to replace convolution operation in the encoder and the decoder. Then we integrate a hybrid attention module containing a spatial attention module and a channel attention module into the network. Finally, due to the high variability in shapes, size and location of the pancreas, we follow Zhou et al. [15] to use a coarse-to-fine framework for pancreas segmentation and apply the proposed network to both coarse and fine phase. In summary, our main contributions are two-fold:

- We propose a novel attention module, which contains a spatial attention module and a channel attention module. The output of these two modules is aggregated to obtain a more robust feature expression.
- We propose a 2.5D network, which combines 2D convolutional layers with 3D convolutional layers and uses 3 adjacent slices to form a 3-channel input image, so that our network can capture the inter-slice information compared with 2D models and needs less computational resources than 3D models. The proposed approach achieves state-of-the-art performance on the NIH pancreas segmentation dataset [9].

## Materials and methods

### Problem definition

In this section, we describe the pancreas segmentation problem as follows. Let *X* ∈ *I*^*n*×*w*×*h*×*c*^ represent slices by cutting along the coronal, sagittal or axial plane, where *n* is the number of slices containing pancreatic region, *h* and *w* represent the width and height of slices, and *c* is the number of channels. Let the binary segmentation mask *Y* ∈ *I*^*n*×*w*×*h*×*c*^ be the annotation of *X*, and it can be defined as follows:

$$\begin{array}{c} Y_{n,w,h,c}=\{\begin{array}{cc} 1, & X_{n,w,h,c}\in pancreas\\ 0, & X_{n,w,h,c}\notin pancreas \end{array} \end{array}$$ (1)

The voxel belongs to the pancreatic region while the value of its corresponding position in *Y* is equal to 1, otherwise the voxel belongs to the background. The pancreas segmentation task is to learn a pixel-wise segmentation model *Seg* to obtain the segmentation map *Seg(X)* of each input image, and our goal is to minimize the difference between the ground truth *Y* and the prediction map $\hat{Y}=Seg\left(X;\theta \right)$, where *θ* indicates the parameters of the model. To evaluate the similarity between the ground truth *Y* and the segmentation map $\hat{Y}$, we follow [11] to use Dice Similarity Coefficient (DSC), which is widely applied to medical image segmentation tasks. DSC is defined as follows:

$$\begin{array}{c} DSC=\frac{2\times |A⋂B|}{\left|A|+|B\right|} \end{array}$$ (2)

where *A* and *B* are two voxel sets. We slightly modify the DSC into a loss function $L(Y,\hat{Y})$, and the segmentation task can be defined as follows:

$$\begin{array}{c} L\left(Y,\hat{Y}\right)=1-\frac{2\times \sum_{i}y_{i}{\hat{y}}_{i}}{\sum_{i}y_{i}+\sum_{i}{\hat{y}}_{i}} \end{array}$$ (3)

where *y*_*i*_ and ${\hat{y}}_{i}$ are the *i*^*th*^ pixels of the segmentation map *Y* and the ground truth $\hat{Y}$, and the value of these pixels are 0 either 1.

### Coarse-to-fine framework

**Fig 2:**
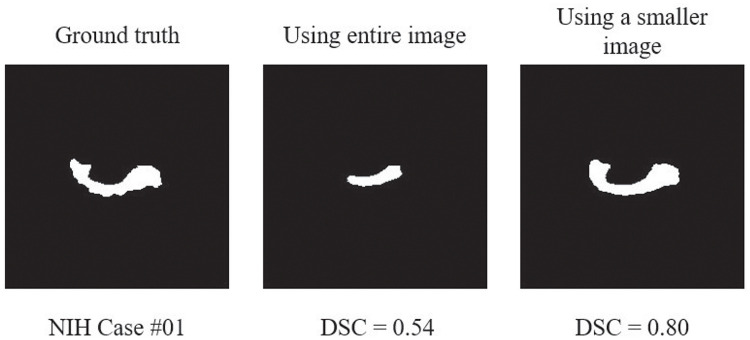
Illustration of the segmentation results of two different input regions. A smaller input region improves the segmentation performance.

The pancreas is an inconspicuous and extremely small organs of irregular shape, which usually occupies less than 2% of the entire CT volume. Furthermore, the pancreas has a high degree of anatomical variability and is indistinguishable from the surrounding organs and tissues, which results in a blurry boundary with other organs and tissues. Therefore, deep neural networks are easily confused by the surrounding organs and the model will bias towards the background. For example, FCN and U-net, which are widely used in semantic segmentation and have an outstanding performance in medical image segmentation, sometimes demonstrate unsatisfactory results for pancreas segmentation task. A smaller input image containing the pancreatic region can efficiently alleviate this problem, see Fig 2.

**Fig 3:**
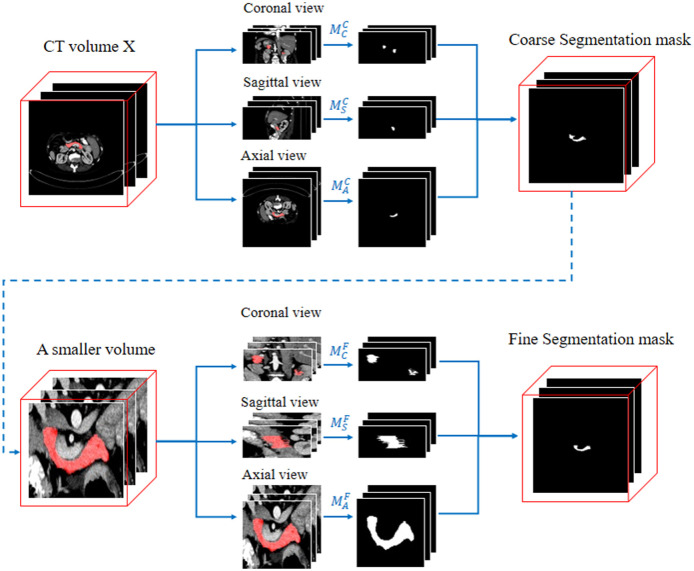
Illustration of the coarse-to-fine framework. The framework segments the pancreas through coronal, sagittal and axial viewpoints and fuses the results into a new segmentation volume.

Inspired by this, we follow Zhou et al. [15] to apply a coarse-to-fine framework (see Fig 3), which crop the entire input image into a smaller input region for the fine stage, according to the prediction masks of the coarse stage. We cut a 3D volume into a set of slices along three axes, *i.e*., the sagittal, axial and coronal views. Then We train two sets of models (each set includes three models, *M*_*s*_, *M*_*a*_ and *M*_*c*_ for three views) for coarse and fine phase. We fuse these prediction masks of three different views into a 3D binary volume via majority vote. The fusion function $r({\hat{Y}}_{s},{\hat{Y}}_{a},{\hat{Y}}_{c})$ can be defined as follows:

$$\begin{array}{c} \hat{Y}=r\left({\hat{Y}}_{s},{\hat{Y}}_{a},{\hat{Y}}_{c}\right)=\frac{{\hat{Y}}_{s}+{\hat{Y}}_{a}+{\hat{Y}}_{c}}{3} \end{array}$$ (4)

where ${\hat{Y}}_{s}$,${\hat{Y}}_{a}$ and ${\hat{Y}}_{c}$ are the segmentation masks of the sagittal, axial and coronal viewpoints, and $\hat{Y}$ is the fusion result.

We add a frame around the coarse segmentation results, which is filled with the origin image, to make the network more robust and avoid over-fitting, then crop the volume into a smaller block.

### Our approach

**Fig 4:**
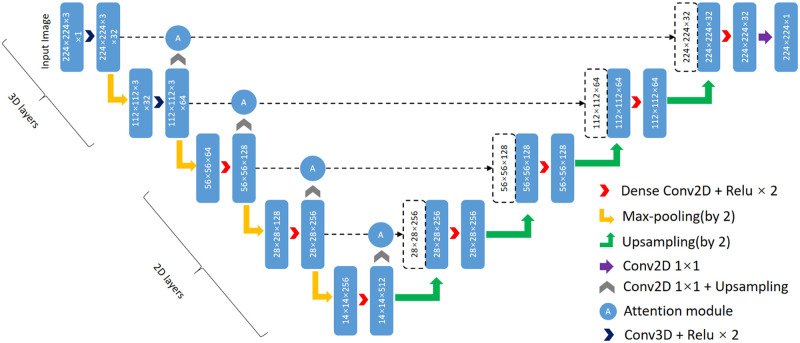
Illustration of the proposed multi-scale attention net. Dense block is show in Fig 5 and the proposed attention module is shown in Fig 6.

In this section, we introduce a novel 2.5D segmentation network in detail. Since U-net has been widely used for medical image segmentation and has achieved outstanding performance, we design a 2.5D U-net architecture (see Fig 4) and integrate a hybrid attention module into it. Currently, 3D segmentation networks need more computational resources and GPU memory, while 2D segmentation models are smaller and less accurate because 2D networks cannot capture spatial information along the third dimension. Therefore, we combine 2D convolutional layers with 3D convolutional layers and use 3 adjacent slices to form a 3-channel input image, so that our approach can extract inter-slice features from these adjacent slices and needs less computational resources than 3D networks. The input image of our approach can be simply described as follows:

$$\begin{array}{c} I_{i}=\{\begin{array}{cc} (S_{i},S_{i},S_{i}), & i=0\;or\;i=n-1\\ (S_{i-1},S_{i},S_{i+1}), & otherwise \end{array} \end{array}$$ (5)

where *S*_*i*_ represents the *i*–*th* slice, and n is the number of slices.

**Fig 5:**
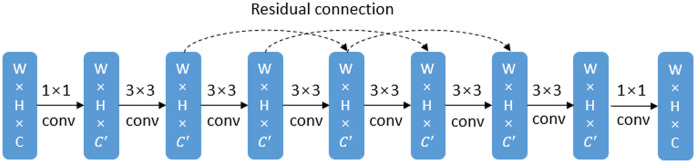
Illustration of the dense block. Dense block is composed of 6 convolution layers to obtain a large receptive field and skip connections are used to avoid the risk of network degradation, gradient vanish and gradient explosion.

Similar to U-net, the proposed architecture includes an encoder structure and a decoder structure, and both of them are composed of 4 blocks. The difference is that for the proposed network, the first two layers of the encoder structure are 3D convolutional layers and the remaining layers are 2D convolutional layers. We use denser convolution layers (see Fig 5) to obtain a larger receptive field, because a larger receptive field is helpful to extract effective features for large object segmentation, while after coarse segmentation the pancreas is relatively a large object compared with the background region. However, denser convolution layers bring about the risk of network degradation, gradient vanish and gradient explosion [25], so we introduce skip connections to alleviate this problem.

Due to the downsampling layers, the proposed network can extract semantic information at different image resolutions, and these feature maps are fused with the feature maps extracted in the decoder structure. In addition to the U-like architecture, a hybrid attention module is integrated into the skip connections between the encoder structure and the decoder structure. This module is composed of a channel attention module and a spatial attention module.

**Fig 6:**
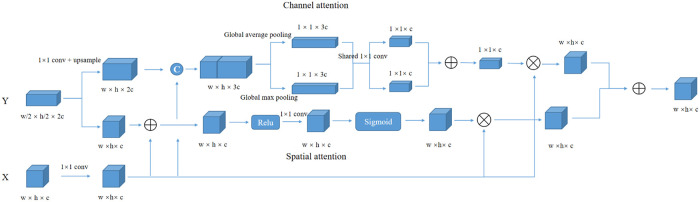
Illustration of the proposed attention module. It is composed of a spatial attention module and a channel attention module, and make the network focus on the most relevant regions and channels.

As is shown in Fig 6, the attention module has two input feature maps *X* ∈ *R*^*w*×*h*×*c*^ and *Y* ∈ *R*^*w*/2×*h*/2×2*c*^ from different dense blocks in the encoder path. For the spatial attention module, input *Y* is upsampled by a factor of 2 and is fused with input *X*, and the position contextual information is obtained by several 1 × 1 convolution layers, then the weight map is obtained through a sigmoid function. Finally, we multiply the weight map and input *X* to get the attention map, and spatial attention can be described as follows:

$$\begin{array}{c} SA=X\sigma (conv(f(X,Y))) \end{array}$$ (6)

where *σ* is the sigmoid function, and *f* represents the fusion layer. For the channel attention module, we concatenate input *X* and input *Y* in the channel dimension, then use the global average pooling layer and global max pooling layer to obtain two 1 × 1 × *c* feature maps, where *c* denotes the number of channels. Average pooling can smoothly extract features but it cannot extract salient features, so that we introduce max pooling to obtain remarkable features of each channel. These two pooling layers are defined as follows:

$$\begin{array}{c} gap=\frac{1}{w\times h}\Sigma_{i}^{w}\Sigma_{j}^{h}I_{c}\left(i,j\right) \end{array}$$ (7)

$$\begin{array}{c} gmp=\max\;I_{c} \end{array}$$ (8)

where *I*_*c*_ denotes *c*^*th*^ channel of input *I*, *w* and *h* are width and height of the input. After a shared convolution layer, a fusion layer and a sigmoid function, we can get the channel weight, then we multiply channel weight and input X to obtain the attention map. Finally, we fuse the spatial attention map with the channel attention map and integrate it into the skip connections between the encoder path and the decoder path. The proposed attention module can help the network focus on the most relevant regions and channels and alleviate the risk of disrupted by the background region of the input image.

## Results

### Dataset

We evaluate our approach on the public NIH pancreas dataset [9], which is the largest and most authoritative Open Source Dataset in pancreas segmentation. The dataset includes 82 contrast-enhanced abdominal 3D CT scans. The resolution of each CT scan is 512 × 512 × *L*, where *L* ∈ [181, 466] is the number of slices along the third axis. The slice thickness varies from 0.5 *mm* to 1.0 *mm*. The pixel values in a CT slice were clipped to [-100, 240], then re-scaled to [0, 255]. We resize the slices to [224, 224] and use 3 adjacent slices to form 3 channels. Following the standard cross-validation strategy, we split the dataset into 4 fixed folds, each of which contains approximately the same number of samples. 3 of 4 subsets are used for training and the remaining one is used for testing. We use DSC to evaluate the similarity between the ground truth and the segmentation mask and record the mean, max, min and standard deviation statistics over all testing cases.

### Implementation details

We implement our approach in Keras framework and train our segmentation model on an NVIDIA GeForce GTX 1060 GPU. Each model of coarse and fine stage is trained for 30 epochs with an Adam optimizer [26]. The learning rate is set to 0.00001 and batch size is set to 1. We use 10% of the training slices as a validation set to check for overfitting. We cut all 3D CT scans along the coronal, sagittal and axial axis and feed these slices into segmentation models to obtain the predication masks. Subsequently, we construct these masks into a 3D volume and then compute the DSC of the whole 3D CT volume. We use slices with more than 100 pixels of pancreatic region for coarse segmentation training.

### Evaluation and analysis

We use the proposed 2.5D U-net for both coarse and fine segmentation. Coarse segmentation results are shown in Table 1, the proposed approach achieves 75.33±7.36, 74.04±9.75 and 76.89±7.44 average DSC for three views and the gap between maximum DSC and minimum DSC is more than 20%. This demonstrates that the network is confused by the background region because of the irregular shape and size of the pancreas. We improve the segmentation accuracy to 78.79±6.83 by fusing these three models via majority voting, which means this fusion operation can make use of the 3D contextual information, to a certain extent.

**Table 1:** Evaluation for coarse segmentation of three axes and fusion segmentation is reported in the table.

Axes	Mean DSC±std	Max DSC	Min DSC
Coronal axis	75.33±7.36	86.22	57.79
Sagittal axis	74.04±9.75	86.17	52.09
Axial axis	76.89±7.44	87.74	59.60
Fusion	78.79±6.83	89.28	68.37

**Fig 7:**
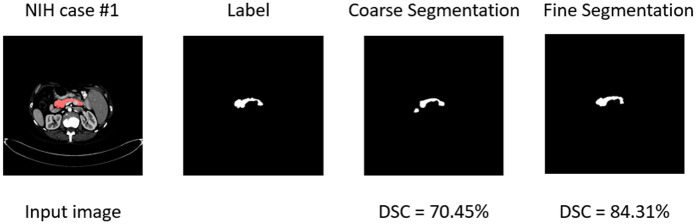
An example of the segmentation results. Compared with the coarse segmentation, fine segmentation result has been improved from 70.45% to 84.31%. However, we can observe that we cannot predict the boundary of the pancreas very well.

Then we evaluate the coarse-to-fine framework on the NIH pancreas dataset and compare the segmentation results of our approach with the state-of-the-art approaches in recent years. The mean, maximum and minimum DSC are presented in Table 2 and an example of the segmentation results is shown in Fig 7. Our segmentation results outperform the best approach [27] by 2%. The minimum DSC has reached 73.89% and the standard deviation has reached 3.47%, which is a significant improvement especially compared with the approach proposed by Roth et al. [28]. This remarkable improvement on minimum DSC suggests that our approach is more stable and reliable on the NIH pancreas dataset. The segmentation accuracy has been significantly improved from 78.79±6.83 to 86.61±3.47, which indicates that a smaller input image can effectively alleviate the risk of being confused by the background region.

**Table 2:** Comparison between our approach and the state-of-the-art approaches on NIH pancreas dataset.

Method	Mean DSC±std	Max DSC	Min DSC
Zhou et al. [15]	82.37±5.86	90.85	62.43
Roth et al. [28]	81.27±6.27	88.96	50.69
Yu et al. [17]	84.50±4.97	91.02	62.81
Asaturyan et al. [29]	79.3±4.4	86.0	72.8
Zhang et al. [27]	84.61±5.21	91.46	70.36
Li et al. [30]	84.19±5.73	91.08	53.61
Our approach	**86.61±3.47**	**92.03**	**73.89**

Segmentation results by ablation study of our approach are demonstrated in Table 3. The results show that our attention module outperforms the attention module proposed by Oktay et al. [24] and Fu et al. [22] and 2.5D U-net architecture achieves better performance. In addition, 2.5D U-net with our attention module costs less time for training phase (about 30 minutes per epoch) than 2.5D U-net with dual attention module [22] (about 70 minutes per epoch). Compared with 2D networks and 3D networks, 2.5D networks achieve higher segmentation accuracy and 2.5D models are much smaller than 3D models.

**Table 3:** Ablation study of our proposed 2.5D U-net and proposed attention module. AG: The attention gate proposed in [24]. DA: The dual attention module proposed in [22]. HA: Our proposed attention module. Params: The number of parameters.

Network	Mean DSC±std	Min DSC	Max DSC	Params
2D U-net	79.50±6.83	56.52	87.94	5.8M
3D U-net	81.37±5.32	60.60	88.90	19.2M
2.5D U-net	81.79±4.88	63.61	89.05	6.9M
2.5D U-net + AG	83.10±5.32	67.36	91.02	7.2M
2.5D U-net + DA	84.07±4.73	67.61	91.08	6.1M
2.5D U-net + HA	86.61±3.47	73.89	92.03	7.3M

We also analyze the DSC distribution of all slices for 2 models. As is shown in Table 4, the number of slices with segmentation accuracy less than 0.5 has decreased from 9.42% to 3.09%, and the number of slices with segmentation accuracy distributing between 0.5 and 0.6 has decreased from 4.92% to 3.70%. This denotes that our approach can effectively address the problem of the background region disruption and is stable on the NIH pancreas dataset.

**Table 4:** Analysis of the DSC distribution of all slices.

Method	U-net	Our approach
DSC [0, 0.5]	9.42%	**3.09%**
DSC (0.5, 0.6]	4.92%	**3.70%**
DSC (0.6, 0.7]	8.94%	10.14%
DSC (0.7, 0.8]	16.96%	18.97%
DSC (0.8, 0.9]	40.24%	44.84%
DSC (0.9, 1]	19.51%	19.26%

## Conclusion

In this paper, we propose a 2.5D U-net and a hybrid attention module for the pancreas segmentation task. The proposed attention module helps the network focus on the relevant region, which alleviate the risk of being confused by the background region. Our 2.5D U-net combines 2D convolutional layers with 3D convolutional layers, and uses 3 adjacent slices to form a 3-channel input image. Therefore, the proposed network achieves a higher accuracy because it can capture the inter-slice information and requires less computational resources than 3D networks. Furthermore, we follow Zhou et al. [15] to apply a coarse-to-fine framework to improve the segmentation accuracy.

Our approach is evaluated on the NIH pancreas dataset, and outperforms state-of-the-art approaches. The minimum DSC for testing cases has also been remarkably improved compared with state-of-the-art approaches, which proves that our approach is stable and can alleviate the risk of being disrupted by the background region.
